# Effects of spinal manipulative therapy biomechanical parameters on clinical and biomechanical outcomes of participants with chronic thoracic pain: a randomized controlled experimental trial

**DOI:** 10.1186/s12891-019-2408-4

**Published:** 2019-01-18

**Authors:** Isabelle Pagé, Martin Descarreaux

**Affiliations:** 1 Department of Anatomy, Université du Québec à Trois-Rivières, Trois-Rivières, Québec, Canada; 2 Neuromusculoskeletal Research Group, Université du Québec à Trois-Rivières, Trois-Rivières, Québec, Canada; 3 Department of Human Kinetics, Université du Québec à Trois-Rivières, Trois-Rivières, Québec, Canada; 4 Present address: Department of Physical Therapy, Faculty of Rehabilitation Medicine, University of Alberta, Edmonton, Alberta Canada

**Keywords:** Spinal stiffness, Thoracic spine, Chronic pain, Electromyography, Spinal manipulation, Manual therapy, Biomechanics, Musculoskeletal manipulation, Back pain, Spine, Dose

## Abstract

**Background:**

Spinal manipulative therapy (SMT) includes biomechanical parameters that vary between clinicians, but for which the influence on the therapy clinical effects is unknown. This parallel-randomized controlled trial aimed to investigate the effect of SMT biomechanical parameters on the outcomes of participants with chronic thoracic pain (CTP) following three treatment sessions (follow-up at one week).

**Methods:**

Adults reporting CTP (pain within the evaluated region [T6 to T8] for ≥3 months) were asked to participate in a four-session trial. At the first session, participants were randomly assigned to one of three experimental groups (different SMT doses) or the control group (no SMT). During the first three sessions, one SMT was executed at T7 for the experimental groups, while a 5-min rest was provided to the control group. SMT were delivered through an apparatus using a servo-controlled linear actuator motor and doses consisted of peak forces, impulse durations, and rates of force application set at 135 N, 125 ms and 920 N/s (group 1), at 250 N, 125 ms and 1840 N/s (group 2), and at 250 N, 250 ms, 920 N/s (group 3). Disability and pain intensity were evaluated at each session (primary outcomes). Spinal stiffness was assessed before-and-after each SMT/rest and at follow-up. Tenderness and muscle activity were evaluated during each spinal stiffness trial. Improvement was evaluated at follow-up. Differences in outcomes between groups and sessions were evaluated as well as factors associated with clinical improvement.

**Results:**

Eighty-one participants were recruited and 17, 20, 20 participants of the three experimental groups and 18 of the control group completed the protocol. In exception of higher pain intensity at baseline in the control group, no between-group differences were found for any of the outcomes. A decrease in pain intensity, disability, spinal stiffness, and tenderness during spinal stiffness were observed (*p*-values< 0.05). At follow-up, 24% of participants were classified as ‘improved’. Predictors of improvement were a greater decrease in pain intensity and in tenderness (*p*-values< 0.05).

**Conclusions:**

In an experimental setting, the delivery of a SMT does not lead to significantly different outcomes in participants with CTP than a control condition (spinal stiffness assessment). Studies are still required to explore the mechanisms underlying SMT effects.

**Trial registration:**

ClinicalTrials.gov NCT03063177, registered 24 February 2017).

**Electronic supplementary material:**

The online version of this article (10.1186/s12891-019-2408-4) contains supplementary material, which is available to authorized users.

## Background

Back pain is highly prevalent in the general population and can lead to important individual and socioeconomic consequences [1]. The annual prevalence of low back pain (LBP) and middle back pain is respectively estimated at around 43 and 35% [2]. Complementary and alternative medicine is widely used by patients with back pain, with about 75% of patients consulting either in chiropractic, physical therapy or osteopathy [3]. Spinal manipulative therapy (SMT) and spinal mobilization constitute treatment options commonly offered by these clinicians and are now recommended in several clinical practice guidelines for the management of spinal pain [4–6]. Overall, these therapies are characterized by the delivery of a force using specific parameters of angulation, amplitude and speed to an intervertebral articulation, which results in specific biomechanical and/or neurophysiological effects [7]. Although SMT targets an intervertebral joint, the manual force is transmitted to the contiguous articulations and surrounding soft tissues. Previous studies have shown the presence of a dose-response relationship between the therapy characteristics and individuals’ neuromechanical responses (i.e. the targeted vertebra displacement, the relative displacement with its adjacent vertebrae and the response amplitude of surrounding muscles) [8–11]. Specifically, the muscle response amplitude increases with the increase in peak force [10], while it decreases when preload forces [9] and the impulse duration increase [8]. Regarding the absolute movement of the contacted vertebra, it increases with increasing peak forces [11] and with the decrease in the preload force [9]. Although SMT yields neuromechanical responses that are believed to be linked to clinical effects, the effect of different SMT doses have been investigated in only one previous randomized controlled trial [12] highlighting the need for further investigations.

Nonspecific back pain regroups heterogenous patients that might not respond similarly to a given treatment. The identification of patient profiles that could help guide treatment options is now recognized as a key issue in back pain research [13]. Recently, monitoring of spinal stiffness measured using a mechanical device has shown to effectively identify patients with LBP who are more susceptible to improve following few SMT sessions [14, 15]. Specifically, Fritz et al. (2011) showed that a decrease in lumbar spinal stiffness following the first treatment is an independent predictor of improved disability at one week after two sessions of lumbopelvic SMT [14]. Using a similar protocol, Wong et al. (2015) observed, in participants with acute and chronic LBP, a significant decrease in L3 spinal stiffness following the first treatment, but only among those who reported a clinically significant improvement in disability following the two treatments [15]. None of these studies have recorded the SMT dose delivered by clinicians nor attempted to standardize the treatment using a mechanical device.

It is therefore unknown if triggering neuromechanical responses of greater magnitude influences SMT clinical, such as pain and disability, and biomechanical, such as spinal stiffness, outcomes in participants with spinal pain. Identifying a dose optimizing these outcomes could have important educational implications and ultimately lead to the improvement of patient care. The overall goal of this study was therefore to increase the understanding of the mechanisms underlying the clinical effects of SMT through an apparatus using a servo-controlled linear actuator motor. Within an experimental paradigm, a modified parallel-randomized controlled trial was designed to investigate the effect of different SMT doses (i.e. including different peak forces and rates of force application) on the clinical and biomechanical outcomes of participants with chronic thoracic pain following three treatment sessions (follow-up one week after the last treatment). It was hypothesized that the SMT peak force and rate of force application would influence the primary (pain intensity and disability) and secondary (spinal stiffness, and tenderness and muscle activity during the assessment of spinal stiffness) outcomes. It was also hypothesized that participants receiving SMT would show a greater improvement in the primary outcomes than those not receiving SMT. Moreover, since this study was exploratory, an analysis of factors associated with clinical improvement was also conducted.

## Methods

### Trial design

This controlled trial used a parallel design where participants were allocated using a 1:1 ratio between the four groups (three experimental groups and one control group). The trial is reported according to the CONSORT 2010 statement [16].

### Participants

Adults with chronic thoracic pain were recruited through advertisements in the Trois-Rivières (Québec, Canada) local newspaper and social media. Inclusion and exclusion criteria are presented in Table 1.

**Table 1:** Inclusion and exclusion criteria

Criteria	Participants with chronic thoracic pain
Inclusion criteria	▪ 18 and 60 years old. ▪ Thoracic pain * for at least 3 months (constant or recurrent). ▪ Pain within T6 to T8 region indicated on the pain diagram and/or during physical examination at the start of the first session. ▪ Pain intensity at the start of the first session ≥5/100.
Exclusion criteria	▪ Having a history of thoracic surgery or fracture. ▪ Diagnosed with a non-spine-related condition that might refers pain to the chest wall (e.g. heart, lung or oesophagus conditions). ▪ Diagnosed or suspected with one of the following conditions: spine-related inflammatory arthritis, aorta aneurism, advanced osteoporosis, neuromuscular disease, myelopathy, malignant tumors, uncontrolled hypertension, radiculopathy, neurologic deficit, thoracic herniated disc, current infection, thoracic scoliosis (Cobb’s angle > 20°). ▪ Being a pregnant woman.

* Thoracic pain was defined as pain in the region bounded superiorly by the T1 spinous process, inferiorly by the T12 spinous process and laterally by the lateral margins of the erector spinae muscles [17]

### Procedures and intervention

**Fig. 1:**
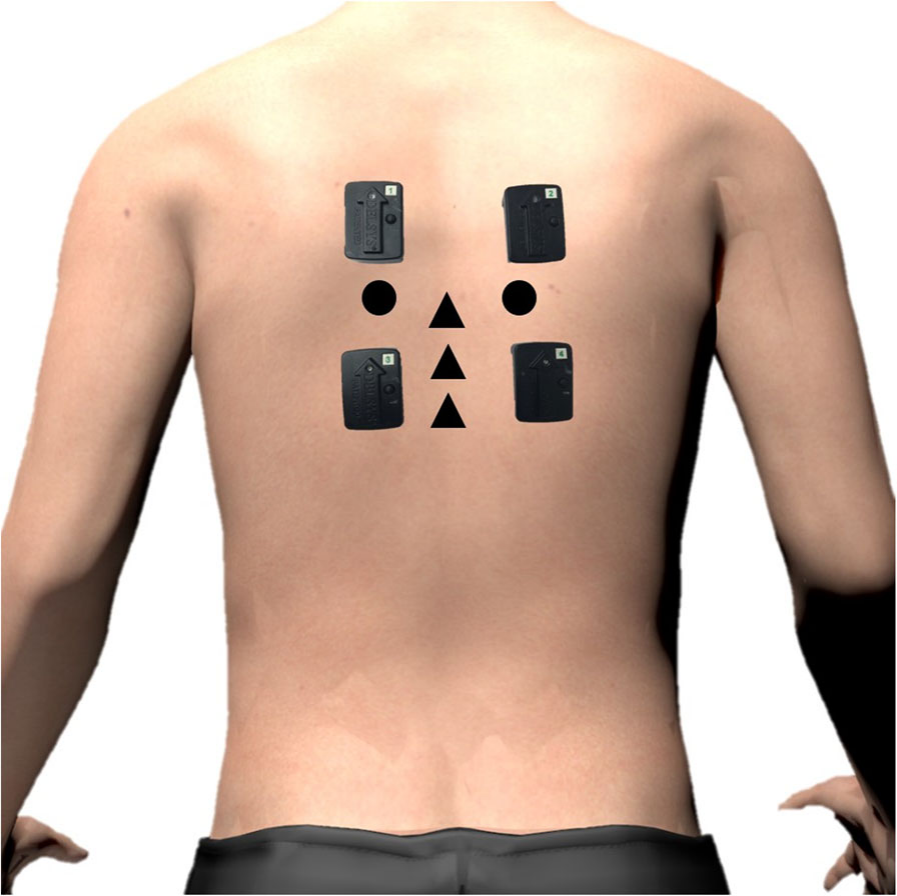
Experimental setup. sEMG electrodes, contact areas during the spinal manipulative therapy (T7 transverse processes; circles) and contact areas during spinal stiffness assessment (T6, T7 and T8 spinous processes; triangles) are visualized

Participants took part in four experimental sessions over a period of 2 to 3 weeks. The first three sessions, that were scheduled two to four days apart, were labeled as the treatment sessions, while the fourth session constituted the follow-up and occurred six to eight days following the last treatment session. A complete description of each procedure is presented below but, briefly, participants first completed a series of questionnaire and were evaluated for inclusion and exclusion criteria (including a physical examination). Following a demonstration of the SMT and spinal stiffness assessment procedures using the apparatus and explanation of its main safety features, participants laid face down on a treatment table (Techniques Tables Ltd., model TT5001029, Ontario, Canada). Identification of T6, T7 and T8 spinous processes and of T7 transverse processes was then performed by the investigator (IP) using a standardized procedure [18, 19] and surface electromyography (sEMG) electrodes were positioned over the thoracic erector spinae just below and over the T7 transverse processes area. A sEMG normalization trial was completed, followed by the assessment of spinal stiffness and SMT (experimental groups) or rest (control group) procedure. Spinal stiffness was reassessed immediately following SMT/rest. These procedures were replicated during the second and third sessions, while the follow-up only included the completion of clinical questionnaires and the assessment of spinal stiffness. Figure 1 shows the experimental setup including the sEMG electrodes localization and contact areas for the assessment of spinal stiffness and SMT delivery.

#### Questionnaires at baseline

At the beginning of the first session, participants’ sex, age, weight and height, in addition to information regarding pain patterns (constant pain about every other day; or recurrent pain less than every other day), sick leave due to thoracic pain and mean pain intensity in the past three months (0–100 visual analog scale with 0 - no pain and 100 – extreme pain; VAS [20]) were gathered. Actual pain intensity (VAS), disability (Quebec Back Pain Disability Questionnaire – QBPDQ, /100) [21], Kinesiophobia (Tampa Scale of Kinesiophobia – TSK, score > 40/68 suggesting kinesiophobia) [22] and risk of symptoms persistence (STarT Back Screening Tool – SBST, score ≥ 5/9 suggesting physical findings accompanied or not by psychosocial barriers to recovery) [23] were also evaluated. Additionally, participants were asked to evaluate their expectation towards their improvement at the fourth session. Participants were considered presenting a positive expectation if they expected that their condition “will improve” or a negative expectation if they expected that their condition “will deteriorate” or “will not change”.

#### Spinal manipulative therapy procedure

**Fig. 2:**
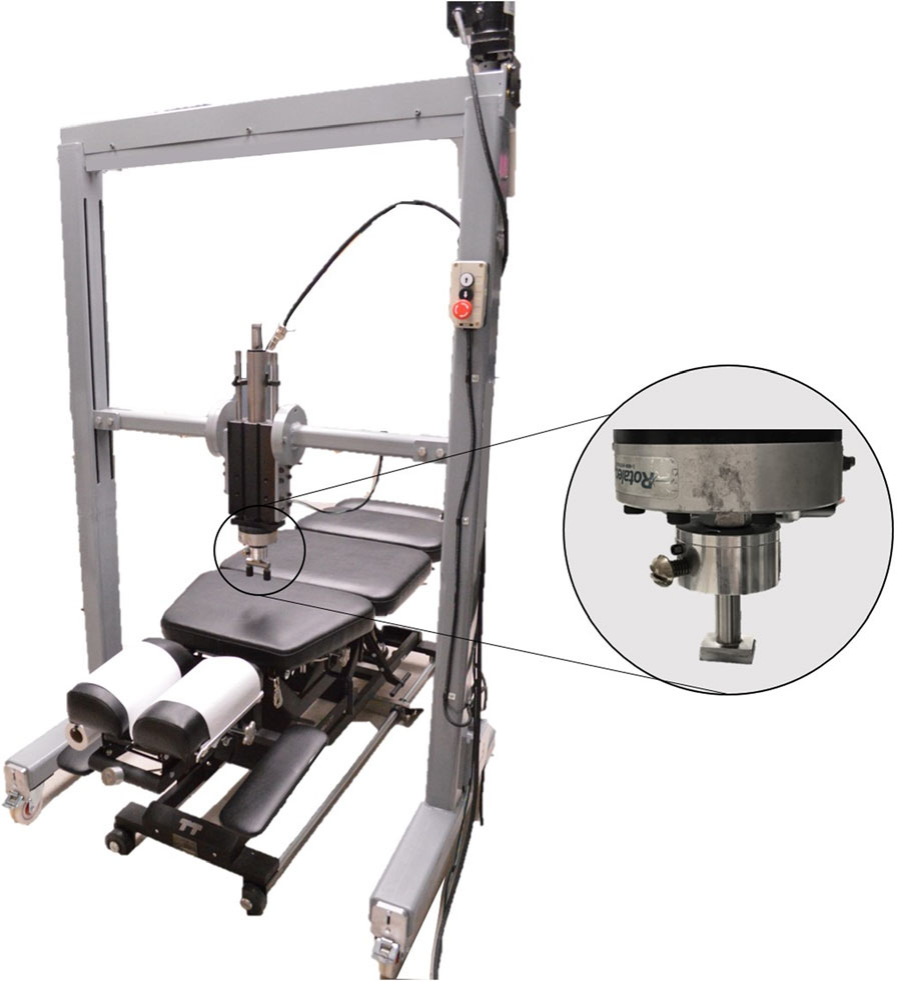
The mechanical device used to deliver the spinal manipulative therapy and to assess spinal stiffness. A twin tip was used during spinal manipulative therapy delivery, while a single tip was used during the assessment of spinal stiffness

The intervention consisted of a single SMT delivered through an apparatus using a servo-controlled linear actuator motor (Linear Motor Series P01-48 × 360, LinMot Inc., Zurich, Switzerland) [24]. The indenter device consisted of a twin-tip padded rod (θ tip = 10 mm; distance between the center of the tips = 56 mm) contacting the skin overlying T7 transverse processes (Fig. 2). This spinal level was targeted for all participants considering technical limitation with the apparatus and the lack of a current gold standard to determine a spinal level that would benefit the most of a SMT. However, participants had to report pain in the targeted area (T6-T8) to be included in the study (see Table 1). SMT were characterized by a preload force of 20 N maintained during 1 s followed by the application of a specific force. The magnitude of the applied force, the impulse duration and the rate of the force application varied between groups (experimental groups) and were within the range of doses used by manual therapists [25]. The *Dose 1* group consisted of a peak force of 135 N applied in 125 ms and resulting in a rate of 920 N/s. These parameters were respectively set at 250 N, 125 ms and 1840 N/s for the *Dose 2* group. The *Dose 3* group consisted of the same peak force as the second group (250 N) applied in 250 ms and resulting in the same rate of force application as the *Dose 1* group (920 N/s). Participants of the fourth group (the control group) rested quietly for 5 min instead of receiving a SMT. For each SMT, the preload force (N), peak force (N), impulse duration (ms), rate of force application (N/s), velocity (mm/s) and indenter displacement during the impulse phase were computed using the mechanical device displacement (mm), force (N) and time (ms) data recorded at 204.8 Hz.

#### Spinal stiffness procedure

Immediately before and after the intervention (SMT/rest), spinal stiffness was assessed four times at T6, T7 and T8 spinous processes by the same apparatus used to deliver the SMT. The measurement of spinal stiffness using this device has been shown to be reliable at T6, T7 and T8 [26]. The indenter head (18 mm × 25 mm) was covered by high-density silicone padding and was positioned over the targeted spinous process (Fig. 2). A randomization scheme [27] was used to determine in which order the spinal levels would be assessed for each participant. For every spinal stiffness trial, the investigator instructed the participant to inhale, exhale, and then hold his breath during measurement (~ 5 s). During exhalations, the linear motor displaced the indenter until applying a 5 N load on the spinous process. While the participant held his breath, a total load of 45 N was gradually applied using an 18 N/s rate of the force application. This load was maintained for 1 s before being withdrawn. LinMot-Talk 5.1 (LinMot Inc., Elkhorn, Wisconsin, USA) was used to sample the applied force and resulting indenter displacement at a frequency of 135 Hz. After each trial, participants rated their tenderness during the procedure (0–100 VAS) and the sEMG activity was recorded during each trial.

#### sEMG procedure

At the beginning of each session, four sEMG electrodes were positioned bilaterally at approximately 2 cm from the spine (over the thoracic erector spinae muscle belly) just above and below the T7 transverse processes. To decrease skin impedance, the skin was shaved, gently abraded with fine-grade sandpaper (Red Dot Trace Prep, 3 M; St. Paul, MN, USA) and cleaned with alcohol swabs. sEMG data were recorded at 2000 Hz using Trigno™ Wireless EMG sensors (Delsys Inc., Natick, Massachusetts, USA). Following instrumentation, participants were asked to perform a normalization trial. This trial consisted in maintaining, without support, the upper trunk in line with the lower body for 5 s. sEMG was recorded during each measurement of spinal stiffness as well as during the impulse phase of each SMT.

### Outcomes

The primary outcomes of this study were pain intensity (VAS) and disability (Quebec Back Pain Disability Questionnaire - QBPDQ), while secondary outcomes were spinal stiffness and both muscle activity and tenderness during the assessment of this parameter. Time points at which outcomes were evaluated are presented in Table 2. The outcome for the exploratory analysis only included the subjective improvement (“improved” or “not improved”) at the follow-up. Indeed, at follow-up, participants completed a subjective overall improvement scale (7-point Likert scale: strongly improved; moderately improved; slightly improved; no change; slightly deteriorated; moderately deteriorated; or strongly deteriorated) [28]. This outcome was further dichotomized in “improved” (moderately or strongly improved) or “not improved” (slightly improved to strongly deteriorated). The outcomes were not modified following the start of the recruitment process and were evaluated by the principal investigator (IP).

**Table 2:** Primary and secondary outcomes evaluated, and time points used for analyses

Outcome	Session 1	Session 2	Session 3	Session 4 (follow-up)
Primary outcomes				
Pain intensity (VAS) at the session beginning	X	X	X	X
Back disability (QBPDQ) at the session beginning	X	X	X	X
Secondary outcomes				
Spinal stiffness at T6, T7 and T8 (N/mm)	Before SMT/rest			X
Muscle activity during spinal stiffness at T6, T7 and T8 (nRMS)	During spinal stiffness procedure before SMT/rest			X
Tenderness during spinal stiffness at T6, T7 and T8 (VAS)	During spinal stiffness procedure before SMT/rest			X
Outcome for the exploratory analysis				
Subjective improvement				X

*VAS* Visual analog scale (0–100); *QBPDQ* Quebec Back Pain Disability Questionnaire (0–100 score); *nRMS* normalized root mean square (i.e. amplitude of the muscle activity)

### Sample size

The required sample size was estimated using G*Power software (G*Power 3.1) based on the results of Haas et al. (2014) that showed an average decrease in pain intensity of 17.70% (SD = 17.35%) in participants with low back pain following 6 weeks of manual therapy [29]. A minimum sample size of 13 participants was determined to detect a statistically significant difference with a power of 0.80, effects size of 0.5 and an alpha value of 0.05. Considering the attrition risk and the comparisons between four groups, a sample size between 15 and 25 participants per group was targeted.

### Randomization

One randomization scheme for males and one for females were generated using an online software [27] by an independent investigator. This investigator subsequently wrote each allocation (either one of the three experimental groups or the control group) in a sealed envelope identified with the participant’s sex (male or female) and a sequential number (1, 2, 3 …). Once a participant provided informed consent, the lead investigator (IP) opened the following envelope of the males’ or females’ pile.

### Blinding

Due to the nature of the intervention, the investigator (IP) and the participants of the control group were not blinded, while participants of the experimental groups were blinded to the specific SMT dose (i.e. the peak force and rate of force application) they received.

### Data analysis

#### Spinal stiffness calculation

To calculate spinal stiffness coefficients, a MATLAB script was developed. Terminal and global spinal stiffness coefficients were calculated using the force and displacement data recorded during each spinal stiffness trial. As previously suggested [30], the first trial of each series of four measurements was excluded. The spinal stiffness value obtained for the second, third and fourth trials were therefore averaged to obtain one terminal and global coefficient for each series of measurement. The terminal coefficient was defined as the ratio of the load divided by the displacement between 10 and 45 N, while the global coefficient was defined as the slope of the straight-line best fitting the data over the same load interval.

#### sEMG data processing

sEMG signals were processed using a custom MATLAB (MathWorks®, Natick, Massachusetts, USA) script. Bipolar sEMG data acquired during normalization trials were first digitally band pass filtered using a 40 Hz low cut-off frequency to filter the electrocardiogram signal contaminating the sEMG signal and a 400 Hz high cut-off frequency (2nd order Butterworth filter). The root mean square (RMS) value was then computed for each electrode during a 2 s time-window, during which the signal was visually stable.

To assess muscle activity during spinal stiffness trials, the sEMG signals were submitted to the same filtering than sEMG signals acquired during the normalization trial. The RMS value was then computed for each electrode between 10 and 45 N application and was normalized (later referred to as nRMS) by dividing it by the respective RMS value obtained during the normalization trial. The average value from the four electrodes was used for subsequent analyzes. The muscle response amplitude during the impulse phase of each SMT was similarly computed.

### Statistical analysis

#### Baseline descriptive and comparative analysis

Mean (with SD) or median (with IQR = Q3-Q1) was computed for the demographic characteristics and clinical questionnaire scores at baseline as well as for spinal stiffness (T6, T7 and T8), muscle activity during spinal stiffness assessment and tenderness during spinal stiffness assessments. Between-group differences were tested using either analysis of variances (ANOVAs) or Kruskal-Wallis tests (for non-parametric data). The neuromechanical responses (displacement and muscle activity) recorded during the first session SMT were also compared between the three experimental groups. The number of participants initially presenting a positive expectation and of those presenting a negative expectation towards their improvement was calculated for each group. Statistics were computed using SPSS Statistics 21 (IBM®, Armonk, New York, USA) and statistical significance was set at *p* ≤ 0.05.

#### Primary outcomes analysis

Since pain intensity (VAS) and disability (QBODQ) were non-normally distributed, they were respectively transformed using the cubic root (VAS_transf_) and the square root (QBPDQ_transf_) to restore quasi-normality. Repeated measures ANOVAs was computed to evaluate if pain intensity (VAS_transf_) and disability (QBPDQ_transf_) at the session beginning differed between sessions and groups, and if an interaction between these variables was present. The presence of a monotonic trend of improvement was determined by computing $\overline{E}$^2^ statistics [31] and Tukey post-hoc tests were computed for significant effects/interactions.

#### Secondary outcomes analysis

Spinal stiffness (terminal and global coefficients), and tenderness (VAS) and muscle activity (nRMS) during the assessment of spinal stiffness were identified as the secondary outcomes of this study. Each coefficient of spinal stiffness was submitted to a 4 × 4 × 3 mixed-model ANOVAs with Tukey post-hoc tests to evaluate if the parameter differed between groups, sessions and spinal levels as well as the presence of interactions between these variables. Since tenderness and muscle activity could not be transformed to meet the distribution requirements of ANOVA, between-group differences were assessed on changes in these variables between the first and fourth sessions using Kruskal-Wallis nonparametric procedure. Moreover, regardless of the group, differences in these variables between the first assessment (pre SMT/rest at the first session) and follow-up (fourth session) were assessed using Wilcoxon Matched-Pairs test.

#### Exploratory analysis of factors associated with clinical improvement

To conduct the exploratory analysis, participants were divided between those “improved” and “not improved” at follow-up. A logistic regression was computed to explore the role of the clinical and biomechanical outcomes in the prediction of “improved” participants. First, differences in the demographic (age, weight, height and BMI), baseline clinical (mean pain intensity in the past 3 months, pain intensity, disability, TSK score, SBST score, and tenderness during spinal stiffness) and biomechanical (muscle activity during spinal stiffness and spinal stiffness) variables were determined between “improved” and “not improved” participants using *t*-test for independent samples (parametric data) or Mann-Whitney U test (nonparametric data). Other potential variables to include in the logistic regression were determined by computing these tests between “improved” and “not improved” participants on the ‘slopes of change’ in VAS_transf_, QBPDQ_transf_, global and terminal spinal stiffness, and tenderness and muscle activity during T6 to T8 spinal stiffness assessment. For each variable, the ‘slope of change’ was calculated between values of the first two sessions and between values of all sessions. The ‘slope of change’ of a specific variable was defined by the coefficient of the straight-line best fitting the values over time for a participant (e.g. pain intensity at session 1, 2, 3 and 4). ‘Slopes of change’ in tenderness, muscle activity and spinal stiffness were also computed between values of before and after the first SMT/rest. All variables presenting significant difference between “improved” and “not improved” participants were included in the logistic regression model. Besides the logistic regression, the positive predictive value of an initial positive expectation and the negative predictive value of an initial negative expectation were computed to explore the role of expectation towards the improvement at follow-up.

## Results

### Recruitment

The recruitment started on May 1st, 2017 and the last follow-up occurred on December 22nd,2017. The trial ended considering that the number of participants within each group laid within the range estimated (15 to 25 per group).

### Participant flow

**Fig. 3:**
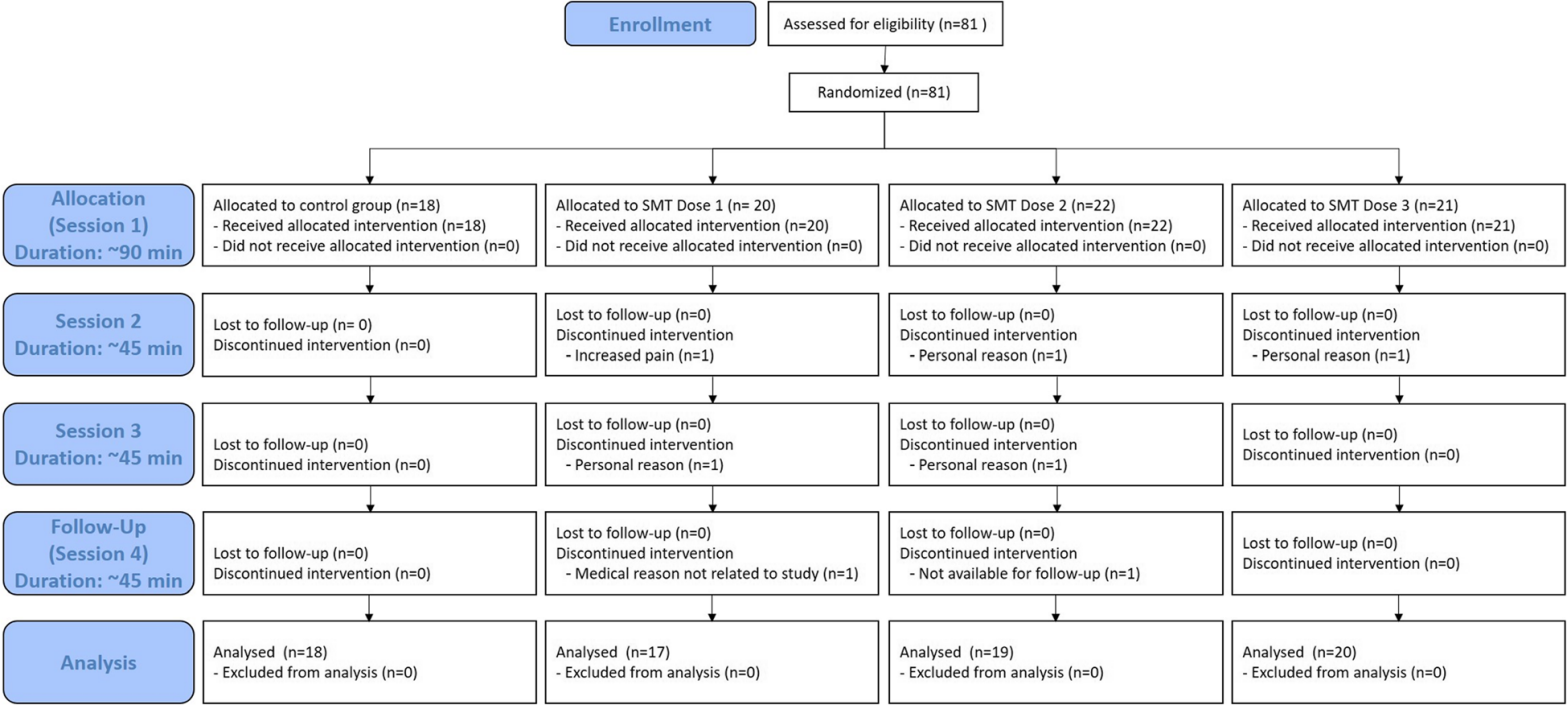
Flow chart of the study. SMT: spinal manipulative therapy

A total of 81 participants were randomly assigned in either one of the three experimental groups (Dose 1 group = 21 participants; Dose 2 group = 22 participants; Dose 3 group = 21 participants) or the control group (18 participants). All participants received the intended intervention. Data of 18 participants of the control group, 17 of the Dose 1 group, 19 of the Dose 2 group and 20 of the Dose 3 group were included for analysis. The experimental flow is shown in Fig. 3.

### Baseline data

Baseline characteristics are reported in Table 3. Groups were similar at baseline with the exception of pain intensity: despite the randomization procedure, participants in the control group presented significantly higher pain intensity at baseline than participants in *Dose 2* group (*p* = 0.01). Initially, 65.0, 81.8, 85.7 and 56.0% of participants in *Dose 1*, *Dose 2*, *Dose 3* and control groups respectively presented a positive expectation towards their improvement at the fourth session.

**Table 3:** Participants’ characteristics at baseline for the experimental groups and the control group

Characteristic		*Dose1* −peak/−rate	*Dose 2* +peak/+rate	*Dose 3* +peak/−rate	Control	*F*_3,77_ or H_3_ value and *p* value
Males: Females		6: 14	8: 14	8: 13	6: 12	–
Age (years)		41.50 (13.79)	37.45 (13.48)	37.19 (11.14)	35.83 (13.68)	*F* = 0.57; *p* = 0.64
Weight (kg)		70.88 (12.66)	72.21 (18.80)	74.66 (18.90)	68.85 (13.15)	*F* = 2.03; *p* = 0.12
Height (m)		1.65 (0.08)	1.67 (0.07)	1.68 (0.10)	1.68 (0.07)	*F* = 0.69; *p* = 0.56
Body mass index (kg/m^2^)		26.12 (4.62)	28.91 (7.15)	26.09 (4.51)	24.48 (4.27)	*F* = 2.43; *p* = 0.07
Average pain intensity in the past three months (0–100, median, IQR)		30.00 (20.00)	27.50 (10.00)	30.00 (26.00)	50.00 (40.00)	H = 7.51; *p* = 0.06
Pain intensity at the start of the first session (0–100, median, IQR)		20.00 (10.00)	20.00 (15.00)	30.00 (29.97)	50.00 (44.00)	H = 12.06, *p* = 0.01*
TSK score (/68)		30.45 (7.26)	31.64 (6.89)	31.71 (8.23)	33.39 (6.17)	*F* = 0.53; *p* = 0.66
QBPDQ score (%, median, IQR)		9.00 (15.00)	16.31 (18.09)	12.92 (11.51)	12.50 (15.00)	H = 1.47 *p* = 0.69
SBST score (/9, median, IQR)		2.00 (3.00)	2.00 (3.00)	3.00 (3.00)	3.50 (2.00)	H = 6.73; *p* = 0.08
Global spinal stiffness at the first session before SMT/rest (N/mm)	T6	8.02 (1.20)	7.49 (1.62)	7.70 (1.62)	8.14 (1.43)	*F* = 0.81; *p* = 0.49
	T7	7.96 (1.05)	7.38 (1.80)	7.72 (1.83)	8.03 (1.18)	*F* = 0.75; *p* = 0.52
	T8	7.73 (1.18)	7.39 (1.87)	7.62 (1.98)	7.80 (1.63)	*F* = 0.23; *p* = 0.87
Terminal spinal stiffness at the first session before SMT/rest (N/mm)	T6	8.06 (1.26)	7.50 (1.65)	7.73 (1.64)	8.20 (1.44)	*F* = 0.88; *p* = 0.46
	T7	7.98 (1.07)	7.41 (1.88)	7.75 (1.95)	8.06 (1.21)	*F* = 0.69; *p* = 0.56
	T8	7.77 (1.24)	7.39 (1.90)	7.64 (2.07)	7.86 (1.66)	*F* = 0.27; *p* = 0.84
Tenderness during spinal stiffness assessment (%, median, IQR)	T6	18.83 (22.50)	13.33 (23.33)	16.67 (18.67)	19.17 (23.33)	H = 2.42; *p* = 0.49
	T7	13.33 (24.33)	5.00 (16.33)	10.00 (20.00)	15.00 (16.33)	H = 2.84; *p* = 0.42
	T8	9.33 (19.33)	4.17 (21.00)	8.33 (16.67)	10.00 (18.33)	H = 1.26; *p* = 0.74
Muscle response amplitude during spinal stiffness assessment (nRMS, median, IQR)	T6	0.10 (0.09)	0.13 (0.11)	0.08 (0.15)	0.11 (0.10)	H = 4.78; *p* = 0.19
	T7	0.10 (0.06)	0.14 (0.13)	0.08 (0.06)	0.11 (0.11)	H = 5.12; *p* = 0.16
	T8	0.09 (0.05)	0.13 (0.13)	0.08 (0.04)	0.11 (0.09)	H = 5.02; *p* = 0.17
Expectation (+: - or neutral)		13: 7	18: 4	18: 3	10: 8	–

Mean and standard deviation are reported unless otherwise indicated

Note: pain intensity and QBPDQ are de-transformed values

* Participants in the control group presented higher pain intensity at baseline than participants in the *Dose 2* group

Abbreviation: *IQR* interquartile range, *TSK* Tampa Scale of Kinesiophobia, *QBPDQ* Quebec Back Pain Disability Questionnaire, *SBST* STarT Back Screening Tool, *SMT* spinal manipulative therapy, *nRMS* normalized root mean square

**Fig. 4:**
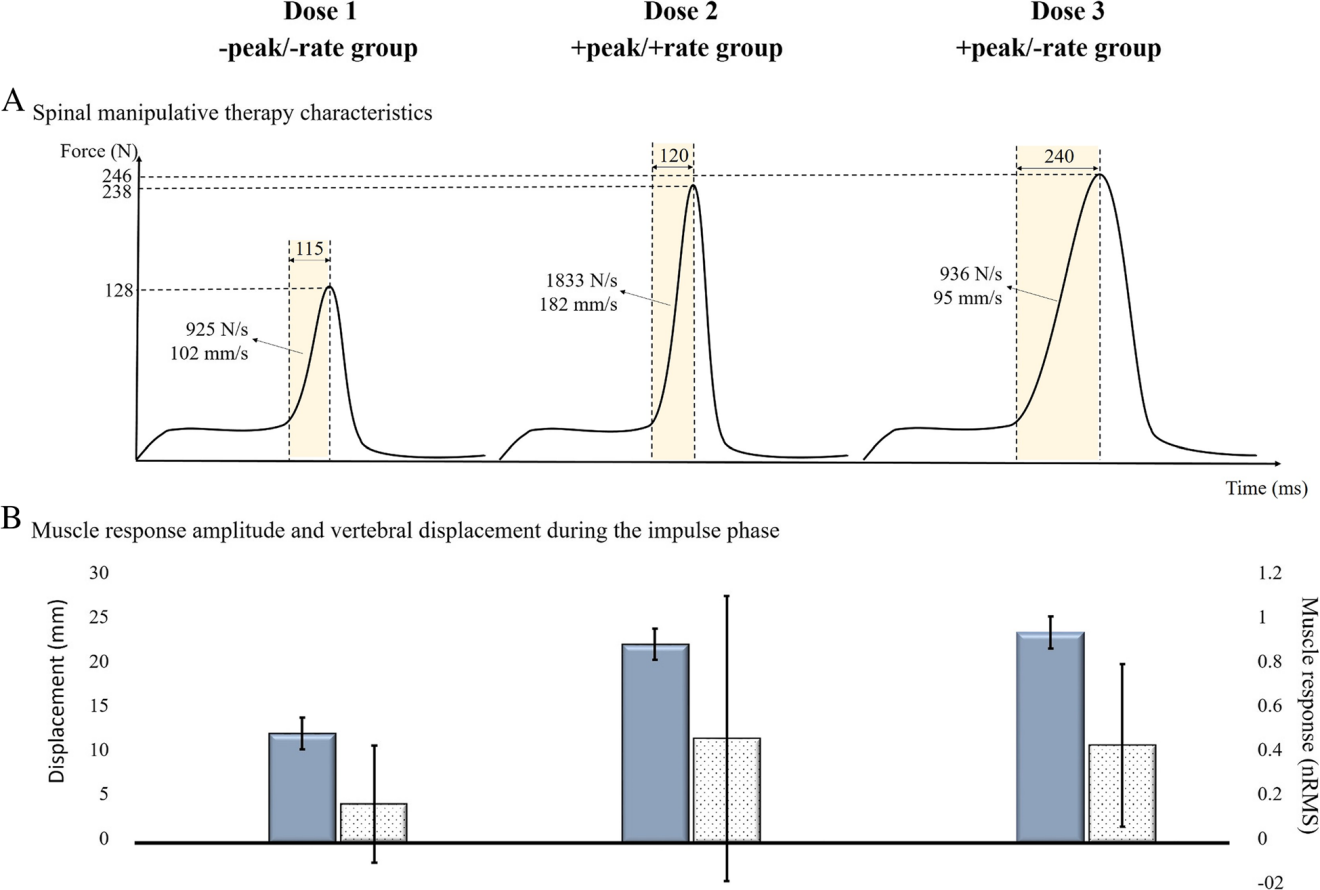
**a** Spinal manipulative therapy parameters, **b** Muscle activity (pale bars, median ± IQR) and indenter displacement (dark bars, mean ± SD)

Comparison of the neuromechanical responses during SMT between the three experimental groups revealed less displacement in the *Dose 1* group (11.82 ± 1.70 mm) than in the *Dose 2* group (21.49 ± 1.69 mm; *p* < 0.001) and the *Dose 3* group (22.70 ± 1.70 mm; *p* < 0.001): F_2, 60_ = 197.30, *p* < 0.001, ${\upeta}_p^2$ = 0.87). Muscle activity during the impulse phase was significantly greater in the *Dose 2* group (median ± IQR = 0.46 ± 0.70) compared to the *Dose 1* group (0.17 ± 0.26; *p* = 0.01): H_2_ = 11.25, *p* = 0.003. Muscle activity during the impulse phase in the Dose 3 group (0.43 ± 0.36) was non-significantly different than the muscle activity in *Dose 1* (*p* = 0.10) and *Dose 2* (*p* = 0.71) groups. These results suggest that the SMT doses generated significantly different neuromechanical responses (displacement and muscle activity). The average value of SMT biomechanical parameters and resulting displacements and muscle activity received by each experimental group may be visualized in the Fig. 4.

### Results for the primary outcomes

Primary and secondary outcomes at each time point are reported in the additional Table (see Additional file 1: Table S1). The analysis revealed no significant between-group differences in disability across the four sessions (F_3, 71_ = 0.43, *p* = 0.73), but confirmed the presence of higher pain intensity at baseline within the control group compared to the *Dose 2* group (F_3, 71_ = 3.61, *p* = 0.02, ${\upeta}_p^2$ = 0.13). Both pain intensity and disability were significantly different between sessions: F_3, 213_ = 18.92, *p* < 0.001, ${\upeta}_p^2$ = 0.21 and F_3, 71_ = 0.43, *p* = 0.73 respectively. $\overline{E}$^2^ statistics confirmed the presence of a gradual improvement across sessions, regardless of group allocation, in regards of pain intensity ($\overline{E}$^2^_4,213_ = 0.21, *p* < 0.01) and disability ($\overline{E}$^2^_4,210_ = 0.33, *p* < 0.01). Pain intensity decreased by an average of − 11.70% (95%CI = − 15.37 to − 7.94) between the first and the fourth session, while disability decreased by an average of − 4.79% (95%CI = − 5.87 to − 3.70). Pain intensity at baseline was also included as a covariable in a repeated-measures ANCOVA for disability but it didn’t impact the results (i.e. no between-group differences but improvement across the sessions).

### Results for the secondary outcomes

Analyses revealed no statistically significant difference in any of the secondary outcomes (spinal stiffness, tenderness and muscle activity during spinal stiffness) between the four groups (all *p* values > 0.05). However, changes over time were observed (including in the control group). Indeed, spinal stiffness was similar at T6 between all sessions (*p values* ≥ 0.05), while, at T7 and T8, spinal stiffness at the first session was significantly higher than at the other sessions (except between the first and third sessions for T7 global stiffness) (*p values* < 0.05). The global stiffness showed a mean decrease of − 0.01 (95%CI = − 0.20 to 0.17) N/mm at T6, − 0.25 (− 0.43 to − 0.07) N/mm at T7 and of − 0.32 (− 0.51 to − 0.13) N/mm at T8. These changes were respectively − 0.04 (− 0.22 to 0.15), − 0.25 (− 0.43 to − 0.08) and − 0.33 (− 0.53 to − 0.14) N/mm for the terminal stiffness. Moreover, a significant decrease in tenderness between the first and the fourth session was observed at T6 (− 3.83, 95%CI = − 6.49 to − 1.17; *z* = 3.18, *p* = 0.001), T7 (− 2.41, 95%CI = − 4.75 to − 0.08; *z* = 2.13, *p* = 0.03) and T8 (− 2.48, 95%CI = − 4.65 to − 0.30; *z* = 2.40, *p* = 0.02). No significant between-session differences were revealed for muscle activity during spinal stiffness (*p* values at each spinal level ≥ 0.05).

### Results for the exploratory analysis

At the fourth session, a total of 18 participants were “improved”, while 59 were “not improved”. Three (16%) participants of the *Dose 1* group, 7 (35%) of the *Dose 2* group, 6 (30%) of the *Dose 3* group and 2 (11%) of the control group were classified as “improved”. No significant differences were observed in the baseline characteristics between “improved” and “not improved” participants (*p* values ≥0.05) with the exception of the SBST score for which “improved” participants showed higher scores than “not improved” participants (U = 366.0, *p* = 0.047). “Improved” participants initially presented a median (IQR, range) SBST score of 4 (3, 1–6), while “not improved” participants presented a median score of 3 (3, 0–8). Probabilities of being “improved” and “not improved” when presenting a positive and a negative expectation were respectively of 28.6% and 90.5%.

Analyses revealed greater ‘slopes of change’ (i.e. a greater decrease across the four sessions or between the first two sessions or between before and after the SMT/rest at the first session) among “improved” participants in 6 variables: pain intensity across the four sessions (U = 268.5, *p* = 0.002); T6 tenderness across the four sessions (U = 326.0, *p* = 0.03); T6 tenderness between the first two sessions (U = 276.0, *p* = 0.002); T8 tenderness between the first two sessions (U = 357.5, *p* = 0.04); T8 terminal stiffness between the first two sessions (U = 359.0, *p* = 0.04); and T7 tenderness between before and after the first SMT/rest (U = 355.0, *p* = 0.03). These variables, as well as SBST score and initial expectation (positive or negative), were entered in the logistic regression to determine if these variables significantly associated with being “improved” at the follow-up (Table 4). Overall association/prediction success was 87.7% (96.4% for “not improved” and 61.1% for “improved” participants): φ = 0.65, χ^2^(1) = 30.61, *p* < 0.001. Overall, a greater decrease in pain intensity across the four sessions (*p* = 0.01), a greater decrease in tenderness during T6 spinal stiffness assessment between the first two sessions (*p* = 0.048), and a higher SBST score (*p* = 0.07) were significant and almost significant predictors of “improved” participants (or significant variables associated with being “improved”).

**Table 4:** Results of the logistic regression analysis to predict “improved” participants

Independent variables	b (SE)	*p*	95% CI for Odds Ratio		
			Lower	Odds Ratio	Upper
Intercept	−3.51 (1.25)	0.01			
STarT Back Screening Tool score	0.43 (0.23)	0.07	0.97	1.53	2.41
Initial expectation: positive vs negative	−1.53 (0.94)	0.11	0.03	0.22	1.37
Slope of the change in pain intensity across the four sessions	0.39 (0.14)	0.01*	1.13	1.48	1.94
Slope of the change in T6 tenderness across the four sessions	0.01 (0.01)	0.50	0.98	1.01	1.04
Slope of the change in T6 tenderness between the first two sessions	0.13 (0.07)	0.048*	1.00	1.14	1.29
Slope of the change in T7 tenderness between before-and-after the first SMT/rest	0.09 (0.15)	0.57	0.81	1.09	1.48
Slope of the change in T8 tenderness between the first two sessions	0.07 (0.05)	0.22	0.96	1.07	1.19
Slope of the change in T8 terminal spinal stiffness between the first two sessions	−1.00 (0.71)	0.16	0.09	0.37	1.48

Note: A positive slope indicates an improvement. R^2^ = 0.36 (Cox & Snell), 0.53 (Nagelkerke). Model χ^2^(8) = 32.02, *p* < 0.001

* Statistically significant predictor/variable

### Harms

One participant within the *Dose 1* group left due increased back pain that subsided more than 72 h after the first SMT.

## Discussion

This study failed to demonstrate an effect of SMT dose (i.e. the peak force and the rate of force application) on the clinical and biomechanical changes in participants with chronic thoracic pain. Overall, a decrease in the primary (pain intensity and disability) and secondary (spinal stiffness and tenderness during spinal stiffness assessment) outcomes were observed across the sessions not only regardless of the therapy dose (groups 1 to 3), but also in the group that did not receive any SMT (control group).

### SMT dose and spine-related pain

Comparisons with previous studies remain limited. The RCT published by Snodgrass et al. (2014) seems to be the only other study comparing the effect of a manual therapy (i.e. spinal mobilization) of different doses on clinical and biomechanical outcomes in a clinical population [12]. These authors evaluated the immediate and the short-term (~ 4 days) change in pressure pain threshold (main outcome) and neck disability, pain intensity, spinal stiffness and range of motion (secondary outcomes) following either a 90 N spinal mobilization, a 30 N spinal mobilization or a placebo (detuned laser) in participants with chronic nonspecific neck pain. Their results revealed no between-group difference on the primary outcome but, in contrast with the current study, suggested a dose effect on neck pain intensity and spinal stiffness. Indeed, the 90 N spinal mobilization group showed, at short-term, greater decrease in pain intensity than the 30 N spinal mobilization group and in spinal stiffness than the placebo group. Authors concluded that a specific dose of mobilization (force applied), appears necessary to reduce spinal stiffness and potentially pain. However, their results also revealed a non-statistically significant change in pain intensity between the 90 N spinal mobilization and the placebo groups, and in spinal stiffness between the 90 N spinal mobilization and the 30 N spinal mobilization groups. These results are therefore more consistent with an absence of a strong influence of SMT characteristics on this therapy effects This might partly explain the lack of significant difference between the groups of the current study especially considering that all participants (included the control group) received light mobilization through the assessment of spinal stiffness.

Besides the fact that the spinal stiffness procedure could have resulted in a clinical improvement and thus might have limited the possibility to detect between-group differences, other reasons could explain the absence of significant between-group differences in the current study. First, although participants were asked to be symptomatic at baseline, the severity of their clinical status could be considered, in average, mild to moderate which could have resulted in a floor effect considering the limited window of improvement. This also explains that the average change in pain intensity (− 11.70%) and disability (− 4.79%) fell below the minimal clinically important difference (MCID) thresholds which are respectively estimated to be 15% and 20% [32]. Future studies should aim to recruit participants presenting at least a moderate level of pain intensity and disability to evaluate the effect of SMT doses in a more clinically relevant population. Secondly, higher pain intensity at baseline was observed in the control group, these participants were, therefore, more susceptible to improve due to the regression toward the mean phenomenon which can have hidden a difference in the clinical improvement between the control group and the experimental groups. Third, it cannot be excluded that the changes in the clinical outcomes reflect the natural improvement of back pain which is known to constitute a cyclic condition and has been reported to be independent of the intervention received [33]. Finally, the improvement might reflect that the contextual factors of a treatment are more important of the treatment modality itself (see the discussion related to the variables associated with improvement).

Regarding the secondary outcomes, it remains difficult to determine if the changes observed between the first and the last sessions are clinically significant. Although statistically significant, the change in tenderness during spinal stiffness remains small and failed to reach the MCID of 15%. The MCID for spinal stiffness is not known but some hypothesis can be raised from the current literature. Latimer et al. (1996) observed an 8% decrease in the initial spinal stiffness when participants reported an improvement of at least 80% in their LBP intensity [34]. This percentage would have represented a decrease of at least 0.60 N/mm in the current study, which is twice the average decrease observed. On the other hand, Wong et al. (2015) showed that, in participants with LBP, a clinical improvement in disability following two treatment sessions is associated to an average decrease in L3 spinal stiffness of 0.26 N/mm (95%CI = 0.08–0.43 N/mm), which is similar to the changes observed in the current study [15]. Finally, in contrast to the observation of an increase in spinal stiffness in participants with LBP [35], a decrease in this parameter has been observed in participants with chronic thoracic pain [26]. It is therefore more likely that these changes in spinal stiffness are not clinically relevant.

### Variables associated with improvement

Considering that people with back pain constitutes a heterogenous population, an exploratory analysis was conducted to identify potential factors associated with a moderate to strong improvement at follow-up. Interestingly, 65% of the 18 “improved” participants received high peak force doses, suggesting that higher peak force increases the probability of improvement in at least some individuals. Current biomechanical and neurophysiological models mostly explained SMT clinical effects by the stimulation of spinal reflexes resulting, among others, in a hypoalgesia effect [36], an increase in spinal mobility [37] and an increase in maximum voluntary contraction and proprioception [38]. However, it cannot be excluded that these clinical effects have, at least partly, been mediated by other factors such as the ones related to the context of treatment [39]. Previous studies showed that participants with neck pain receiving SMT and initially believing that this therapy would help them were more likely to improve in comparison to participants not considering this therapy as potentially beneficial [29, 40]. In the current study, 90.5% of participants initially presenting a negative expectation towards the treatment effect were, indeed, “not improved” at the fourth session. Noteworthy, participants with negative expectations were twice as many in the control and low peak force dose groups than in the high peak force dose groups. The portion of the clinical improvement associated with these nonspecific effects is not known, but these results highlight the importance of considering patient preferences and expectations in the choice of treatment modalities. Interestingly, previous studies failed to identify a prognostic value for the STarT Back Screening Tool in chiropractic settings [41]. In contrast to the studies included in the narrative review of Khan (2017), the 0–9 score was used in the current study instead of the 3-level risk stratification suggested by Hill et al. (2008) [42]. A score of > 3/9 on the SBST suggests the presence of physical findings that needs to be tailored, which can be accompanied or not by psycho-social barriers to recovery [42]. The reason why individuals with higher SBST score were more likely to be “improved” is not known but such results certainly warrants further investigation. The exploratory analysis also supports previous literature on the importance of response to first treatments [43, 44]. Indeed, a greater slope of change in pain intensity across the four sessions and a greater slope of change in tenderness during spinal stiffness at the targeted vertebra were predictors of a moderate to strong improvement. Considering that patients’ improvement at the fourth session was shown to be a strong predictor of patient improvement at 3 and 12 months [45], importance of monitoring patient changes in pain intensity and tenderness across the first treatments to determine if a specific management is appropriate needs to be investigated.

### Neuromechanical responses and SMT

The current results do not support the hypothesis that SMT characteristics influence the clinical effects of this therapy. Indeed, the comparisons of the neuromechanical responses between the three experimental groups confirmed that different responses were triggered although no between-group differences were revealed. Greater absolute vertebral displacement (indirectly obtained by the indenter displacement) was recorded in the two high peak force doses compared to the low peak force dose. Relative displacements were not measured in the current study, but SMT of lower peak force would unavoidably result in a lower relative displacement compared to SMT of greater peak force when delivered with a similar rate of force application. Although it was initially hypothesized that the two high rate of force application doses would show similar and greater muscle response than the low rate dose, the only significant difference was observed between the high peak force/high rate dose and the low peak force/low rate dose. Whether a certain threshold of either neuromechanical responses is required to produce a clinical effect in participants with back pain can’t be determined with the design of the current study but should be considered in future studies.

### Strengths and limitations

This study provides the first preliminary data regarding the influence of SMT characteristics on the therapy clinical/biomechanical effects using an RCT. The main strength is the between-group comparisons using a randomization sequence to minimize differences between them. Unfortunately, despite randomization, between-group differences in baseline pain intensity were present which may have influenced the results. Moreover, this study used a mechanical device to deliver SMT and to assess spinal stiffness, which minimized between-participants and between-day variations. In contrast to previous studies evaluating SMT clinical effects, the therapy characteristics and the size of the contact surface were controlled in the current study. Moreover, a low attrition rate was reached (i.e. 7%).

Some limitations also need to be considered when interpreting the results of the current study. First, the interventions were not delivered within a clinical setting; therefore, the effects related to the clinician-patient relationship, the SMT modulation during its delivery through feedback mechanisms, and to other components of patient management have not been evaluated. This constitute both a strength and a limitation of the study since it allowed the evaluation of the effect related to the SMT itself but did not capture the whole effect of a management involving this therapeutic modality. Secondly, in average, participants initially presented levels of pain intensity and disability that can be considered as being low to moderate. Results should therefore should be generalized with caution to other populations and future studies should recruit participants with a more clinically relevant status. It must also be noted that only three doses were evaluated. These doses, selected based on the previously conducted studies with the same apparatus [8–11], do not represent the whole and neither the average range of values performed by clinicians. Moreover, to ensure safety, peak forces remained in the lower/mid-range of forces reported in the literature which imply lower dose than the ones that could be delivered to certain patients within a clinical setting (e.g. for a large or fit [wo]man). It is consequently relevant to conduct studies comparing other doses or evaluating associations between SMT characteristics performed by clinicians and the clinical improvement of patients. Finally, considering that the therapy characteristics and the treated area were not tailored to the participant’s morphology and complaints, future studies should investigate whether a personalized/targeted treatment can optimize clinical effects as well as if a participant not improving following a SMT dose could improve with a different one.

## Conclusion

This study showed, that in an experimental setting, the delivery of a SMT does not lead to significantly different outcomes (clinical and biomechanical) in participants with chronic thoracic pain than a control condition only including the evaluation of spinal stiffness. A decrease in pain intensity, disability, spinal stiffness and tenderness during spinal stiffness assessment following four experimental sessions was observed regardless of the group allocation. Studies are still required to explore the mechanisms underlying SMT clinical effects and to identify key characteristics of patients rapidly improving with this therapeutic modality. Overall, the observation of a rapid decrease in pain intensity and in tenderness during pressures over the spinous processes are performed constitute a better indicator of treatment success than the treatment characteristics.

## Additional files

Additional file 1: Table S1. Primary and secondary outcomes at the different time points. (DOCX 19 kb)

Additional file 2: Anonymised data to replicate the analyses. (XLS 161 kb)

## Abbreviations

BMI: Body mass index
IQR: Interquartile range
GS: Global coefficient
LBP: Low back pain
MCID: Minimal clinically important differences
QBPDQ: Quebec Back Pain Disability Questionnaire
RMS: Root mean square
SBST: STarT Back Screening Tool
sEMG: Surface electromyography
SMT: Spinal manipulative therapy
TS: Terminal coefficient
TSK: Tampa Scale of Kinesiophobia
VAS: Visual analog scale
