# Supplementary material for: Effects of spinal manipulative therapy biomechanical parameters on clinical and biomechanical outcomes of participants with chronic thoracic pain: a randomized controlled experimental trial
Source: BMC Musculoskelet Disord. 2019 Jan 18;20:29. doi: 10.1186/s12891-019-2408-4 (PMC6339327; doi:10.1186/s12891-019-2408-4)
Supplement: Supplementary file 1 — Table S1. Primary and secondary outcomes at the different time points. (DOCX 19 kb) [file 12891_2019_2408_MOESM1_ESM.docx]

**Additional table. Primary and secondary outcomes at the different time points.**

|  | **Session** | **Dose 1 group** | **Dose 2 group** | **Dose 3 group** | **Control group** | **All participants** |
| --- | --- | --- | --- | --- | --- | --- |
| **Main outcomes (detransformed value with -SD and +SD)** | | | | | | |
| Pain intensity (0-100 VAS) | N | 17 | 20 | 20 | 18 | 75 |
|  | 1 | 21.25 (9.96 – 38.91) | 18.61 (8.29 – 35.16) | 31.55 (16.27 - 54.27) | 39.30 (21.11 - 65.76) | 27.00 (13.72 - 46.90) |
|  | 2 | 16.00 (4.87 – 37.42) | 10.94 (2.83 – 27.68) | 21.25 (7.59 – 45.69) | 23.64 (8.79 – 49.68) | 17.58 (5.21 – 41.64) |
|  | 3 | 16.39 (3.44 – 45.53) | 9.94 (1.41 – 32.11) | 15.07 (2.99 – 42.82) | 21.95 (5.66 – 55.67) | 15.44 (3.05 – 43.96) |
|  | 4 | 10.94 (1.51 – 35.68) | 5.18 (0.28 – 22.03) | 13.65 (2.28 – 41.54) | 22.19 (4.97 – 59.92) | 12.01 (0.74 – 49.66) |
| Disability (0-100) | N | 17 | 20 | 20 | 18 | 75 |
|  | 1 | 10.76 (2.84 - 23.75) | 13.40 (4.27 – 27.60) | 14.90 (5.14 – 29.74) | 14.52 (4.91 – 29.20) | 13.32 (4.37 – 27.13) |
|  | 2 | 8.12 (1.25 – 20.99) | 9.73 (1.93 – 23.54) | 11.62 (2.82 – 26.44) | 9.86 (1.98 – 23.73) | 9.80 (1.95 – 23.64) |
|  | 3 | 6.60 (0.71 – 18.48) | 8.58 (1.44 – 21.71) | 10.05 (2.08 – 24.00) | 9.42 (1.80 – 23.03) | 8.64 (1.46 – 21.83) |
|  | 4 | 6.05 (0.59 – 17.25) | 8.47 (1.48 – 21.19) | 9.06 (1.73 – 22.12) | 9.73 (2.03 – 23.17) | 8.24 (1.29 – 21.18) |
| **Secondary outcomes** | | | | | | |
| Global stiffness (N/mm; mean ± SD) | N | 17 | 19 | 20 | 18 | 74 |
|  | 1 | T6 = 7.97 ± 1.48  T7 = 7.89 ± 1.51  T8 = 7.60 ± 1.69 | T6 = 7.34 ± 1.48  T7 = 7.27 ± 1.51  T8 = 7.27 ± 1.69 | T6 = 7.65 ± 1.48  T7 = 7.64 ± 1.51  T8 = 7.48 ± 1.69 | T6 = 8.14 ± 1.48  T7 = 8.03 ± 1.51  T8 = 7.80 ± 1.69 | T6 = 7.78 ± 1.48  T7 = 7.71 ± 1.51  T8 = 7.54 ± 1.69 |
|  | 2 | T6 = 7.54 ± 1.50  T7 = 7.29 ± 1.47  T8 = 7.30 ± 1.53 | T6 = 7.35 ± 1.50  T7 = 7.07 ± 1.47  T8 = 6.90 ± 1.53 | T6 = 7.61 ± 1.50  T7 = 7.61 ± 1.47  T8 = 7.34 ± 1.53 | T6 = 8.13 ± 1.50  T7 = 7.94 ± 1.47  T8 = 7.69 ± 1.53 | T6 = 7.66 ± 1.50  T7 = 7.48 ± 1.47  T8 = 7.31 ± 1.53 |
|  | 3 | T6 = 7.72 ± 1.50  T7 = 7.49 ± 1.51  T8 = 7.37 ± 1.55 | T6 = 7.36 ± 1.50  T7 = 7.15 ± 1.51  T8 = 6.92 ± 1.55 | T6 = 7.51 ± 1.50  T7 = 7.53 ± 1.51  T8 = 7.17 ± 1.55 | T6 = 8.19 ± 1.50  T7 = 7.89 ± 1.51  T8 = 7.62 ± 1.55 | T6 = 7.69 ± 1.50  T7 = 7.51 ± 1.52  T8 = 7.27 ± 1.56 |
|  | 4 | T6 = 7.54 ± 1.42  T7 = 7.25 ± 1.57  T8 = 7.20 ± 1.56 | T6 = 7.50 ± 1.42  T7 = 7.04 ± 1.57  T8 = 6.93 ± 1.56 | T6 = 7.82 ± 1.42  T7 = 7.62 ± 1.57  T8 = 7.29 ± 1.56 | T6 = 8.12 ± 1.42  T7 = 7.86 ± 1.57  T8 = 7.43 ± 1.56 | T6 = 7.74 ± 1.42  T7 = 7.44 ± 1.57  T8 = 7.21 ± 1.56 |
| Terminal stiffness (N/mm; mean ± SD) |  | 17 | 19 | 20 | 18 | 74 |
|  | 1 | T6 = 8.03 ± 1.51  T7 = 7.92 ± 1.58  T8 =7.63 ± 1.74 | T6 = 7.36 ± 1.51  T7 = 7.28 ± 1.58  T8 = 7.26 ± 1.74 | T6 = 7.68 ± 1.51  T7 = 7.67 ± 1.58  T8 = 7.51 ± 1.74 | T6 = 8.20 ± 1.51  T7 = 8.06 ± 1.58  T8 = 7.86 ± 1.74 | T6 = 7.82 ± 1.51  T7 = 7.73 ± 1.59  T8 =7.56 ± 1.75 |
|  | 2 | T6 = 7.57 ± 1.55  T7 = 7.30 ± 1.51  T8 = 7.34 ± 1.56 | T6 = 7.34 ± 1.55  T7 = 7.11 ± 1.51  T8 = 6.90 ± 1.56 | T6 = 7.62 ± 1.55  T7 = 7.65 ± 1.51  T8 = 7.35 ± 1.56 | T6 = 8.15 ± 1.55  T7 = 7.94 ± 1.51  T8 = 7.71 ± 1.56 | T6 = 7.67 ± 1.55  T7 = 7.50 ± 1.51  T8 = 7.32 ± 1.56 |
|  | 3 | T6 = 7.76 ± 1.54  T7 = 7.47 ± 1.56  T8 = 7.37 ± 1.60 | T6 = 7.39 ± 1.54  T7 =7.17 ± 1.56  T8 = 6.88 ± 1.60 | T6 = 7.48 ± 1.54  T7 = 7.53 ± 1.57  T8 = 7.20 ± 1.60 | T6 = 8.22 ± 1.54  T7 = 7.88 ± 1.56  T8 = 7.68 ± 1.60 | T6 = 7.71 ± 1.54  T7 = 7.51 ± 1.56  T8 = 7.28 ± 1.60 |
|  | 4 | T6 = 7.60 ± 1.47  T7 = 7.29 ± 1.61  T8 = 7.20 ± 1.60 | T6 = 7.52 ± 1.47  T7 = 7.05 ± 1.61  T8 = 6.95 ± 1.60 | T6 = 7.82 ± 1.47  T7 = 7.65 ± 1.61  T8 = 7.33 ± 1.60 | T6 = 8.13 ± 1.47  T7 = 7.90 ± 1.61  T8 = 7.42 ± 1.60 | T6 = 7.77 ± 1.47  T7 = 7.47 ± 1.61  T8 = 7.22 ± 1.60 |
| Tenderness during spinal stiffness (0-100 VAS; median ± IQR) | N | 17 | 19 | 20 | 18 | 74 |
|  | 1 | T6 = 18.33 ± 18.33  T7 = 11.67 ± 15.00  T8 = 8.67 ± 15.00 | T6 = 13.33 ± 21.67  T7 = 5.00 ± 14.33  T8 = 5.00 ± 21.00 | T6 = 16.67 ± 19.33  T7 = 10.00 ± 25.00  T8 =6.67 ± 18.33 | T6 = 19.17 ± 23.33  T7 = 15.00 ± 16.33  T8 = 10.00 ± 18.33 | T6 = 16.67 ± 19.67  T7 = 10.00 ± 16.67  T8 = 6.33 ± 17.67 |
|  | 4 | T6 = 16.00 ± 19.33  T7 = 10.00 ± 17.00  T8 = 6.67 ± 20.00 | T6 = 5.67 ± 18.00  T7 = 1.33 ± 8.33  T8 = 0.33 ± 8.33 | T6 = 18.33 ± 20.67  T7 = 9.17 ± 23.17  T8 = 1.17 ± 15.83 | T6 = 13.00 ± 24.00  T7 = 8.67 ± 23.33  T8 = 8.33 ± 18.00 | T6 = 15.00 ± 20.67  T7 = 5.83 ± 20.67  T8 = 3.33 ± 15.00 |
| Muscle activity during spinal stiffness (nRMS; median ± IQR) | N | 17 | 17 | 20 | 16 | 70 |
|  | 1 | T6 = 0.09 ± 0.08  T7 = 0.10 ± 0.04  T8 = 0.08 ± 0.07 | T6 = 0.14 ± 0.11  T7 = 0.14 ± 0.14  T8 = 0.16 ± 0.12 | T6 = 0.08 ± 0.16  T7 = 0.08 ± 0.11  T8 =0.08 ± 0.10 | T6 = 0.11 ± 0.10  T7 = 0.11 ± 0.11  T8 = 0.11 ± 0.09 | T6 = 0.10 ± 0.10  T7 = 0.10 ± 0.12  T8 = 0.10 ± 0.10 |
|  | 4 | T6 = 0.08 ± 0.07  T7 = 0.09 ± 0.08  T8 = 0.10 ± 0.07 | T6 = 0.14 ± 0.23  T7 = 0.15 ± 0.20  T8 = 0.15 ± 0.23 | T6 = 0.10 ± 0.09  T7 = 0.09 ± 0.10  T8 = 0.10 ± 0.09 | T6 = 0.12 ± 0.06  T7 = 0.11 ± 0.07  T8 = 0.11 ± 0.11 | T6 = 0.11 ± 0.10  T7 = 0.10 ± 0.10  T8 = 0.10 ± 0.11 |
